# The principles of whole-hospital predictive analytics monitoring for clinical medicine originated in the neonatal ICU

**DOI:** 10.1038/s41746-022-00584-y

**Published:** 2022-03-31

**Authors:** J. Randall Moorman

**Affiliations:** Cardiovascular Division, Department of Internal Medicine, Center for Advanced Medical Analytics, University of Virginia, Charlottesville, VA USA

**Keywords:** Diagnosis, Biomedical engineering

## Abstract

In 2011, a multicenter group spearheaded at the University of Virginia demonstrated reduced mortality from real-time continuous cardiorespiratory monitoring in the neonatal ICU using what we now call Artificial Intelligence, Big Data, and Machine Learning. The large, randomized heart rate characteristics trial made real, for the first time that we know of, the promise that early detection of illness would allow earlier and more effective intervention and improved patient outcomes. Currently, though, we hear as much of failures as we do of successes in the rapidly growing field of predictive analytics monitoring that has followed. This Perspective aims to describe the principles of how we developed heart rate characteristics monitoring for neonatal sepsis and then applied them throughout adult ICU and hospital medicine. It primarily reflects the work since the 1990s of the University of Virginia group: the theme is that sudden and catastrophic deteriorations can be preceded by subclinical but measurable physiological changes apparent in the continuous cardiorespiratory monitoring and electronic health record.

## Introduction

“When once the diseased skin begins to swell, you will see men asking in vain for treatment.

Meet the disease on its way to attack you.” Persius, *Satires* III^1^

The dream of anticipatory medicine is tantalizing but largely unrealized. We have pressing needs: patients are not only more numerous but also more ill, representing a population that would have died with usual care a decade or two ago. We also have expanding opportunities: clinical data are much more voluminous these days, presenting themselves in greater and greater variety and at higher and higher velocity. Unchanged, though, is the nature of day-to-day patient management: more than ever before, we spend our time reacting to in-the-moment catastrophic clinical deteriorations. While experienced clinicians agree that subtle premonitory changes can be apparent to the right eyes, automated detection of deterioration through sophisticated analysis of already available data is yet to transform the day-to-day practice of medicine. This notion, which we call *predictive analytics monitoring*, has been under fire recently: witness the poor performances of IBM Watson^2^ and the Epic Sepsis Model^3,4^, and the finding that the “predictive” power of the electronic health record (EHR) lies mainly in what the physicians ordered, not results from the patients^5^.

We fear that the haste to generate academic and commercial products has diverted focus toward the electronic health record (EHR)—a blurry image, at best, of the bedside—and away from the doctor, the nurse, the patient, and the continuous cardiorespiratory monitoring. Moreover, we believe that clinical points of view from the bedside have been subjugated to the perceived need for Big Data, so Big that the resolution of clinical definitions is lost. In order to realize fully the potential benefits of hospital-wide predictive analytics monitoring, we argue for a return to original principles, emphasizing clinical experience and reasoning, comprehensive and well-resolved data, sound mathematics, and the nuanced rigor of real-world practice.

## The heart rate characteristics monitoring trial

In 2011 we published the results of one of the largest individually randomized clinical trials ever undertaken in premature infants^6^. Previously, we found that premature infants who are early in the course of sepsis often have abnormal heart rate characteristics of reduced heart rate variability and transient decelerations^7^. We developed or adapted mathematical time-series analytics that reflected the degree to which these abnormalities were present^8–11^ and mapped them to the probability of sepsis in the next 24 h. We developed a logistics regression model adjusted for repeated measures and externally validated it at Wake Forest University^12^.

In the trial, we found that displaying a risk estimate based only on continuous cardiorespiratory monitoring streaming from the bedside monitors led to a more than 20% reduction in mortality^6^. The only intervention was the display of the changing risk of sepsis: there were no alerts, alarms, thresholds, or mandated actions. The clinical benefits—lives saved, length of stay reduced^13^, neurodevelopmental problems decreased^14^—have been durable^15^. The mechanism was as intended—infants with sepsis were saved^16^.

## Principles underlying the development of predictive analytics monitoring

The heart rate characteristics monitoring trial was one of the very earliest and most emphatic proofs of a general principle: predictive analytics monitoring saves lives by detecting subacute potentially catastrophic illness. Table 1 recalls the properties we sought and the questions we asked when we developed predictive analytics monitoring for neonatal sepsis 1.

**Table 1:** Properties and key questions for predictive analytics monitoring research and development.

Property	Key question
Clinical fit	If we detect the problem early, can we do something about it?
Face validity	Can we expect a subclinical prodrome detectable early on?
Signature of illness	Do we record the right signals?
Mathematical foundations	Do we analyze those signals in the right way?
Ground truth events	Is the model trained on the complete, undiluted set of actual cases?
Dynamicity	Does the risk estimate rise as the disease nears?
Clinical trial	Does it work in real life?

### Clinical fit

Sepsis is a common and potentially catastrophic illness, especially in premature infants where it greatly increases morbidity and mortality. The diagnosis is elusive because it presents with non-specific findings, but delaying antibiotics increases the death rate^17^. The need for earlier detection has long been called for by authoritative groups such as the Neonatal Research Network of the NICHD^18^.

Throughout the hospital, in fact, subacute, potentially catastrophic illnesses are common and have adverse outcomes. For example, we found that more than 10% of patients in a surgical and trauma ICU had at least one such event and that the impact on outcomes was outsized: several-fold increases in length of stay and even larger fold increases in death rates^19^. Further, ward patients who deteriorated clinically and were transferred to ICUs had a 40-fold increase in mortality^20^.

#### Perspective

Predictive analytics monitoring fits well clinically. It can meet a need for improved care in conditions where early detection might lead to earlier treatment which, in turn, might reasonably be expected to improve outcomes.

### Face validity

Though it may present suddenly, sepsis in infants (and children^21^ and adults)^19^ is not a sudden illness, so we expect premonitory changes. When clinicians look back on septic patients for whom we made the diagnosis late, we can see subtle but consistent findings of rising heart rates, falling blood pressures, changing temperatures, and white blood cell counts.

While sepsis is a flagship example, there are other subacute potentially catastrophic illnesses in which we can expect a subclinical prodrome. These include respiratory deterioration leading to emergency intubation^19,22,23^, hemorrhage leading to large transfusion^19,24,25^, hypoglycemia^26^, and the multiple reasons that ward patients deteriorate and require ICU transfer^20,27^. Their common characteristics are (1) a natural progression of physiological derangement that begins subtly, (2) a logical approach to diagnostic testing, and (3) therapy that is most effective early in the course of the illness. In our examples above, these include chest X-rays, bronchodilators, diuretics, antibiotics; or angiography, blood counts, surgery, transfusions; or fingersticks, feedings, glucose; or any of the many tests and treatments for the diverse and idiosyncratic modes of clinical deterioration. In each case, an early start to diagnosis and treatment seems likely to help some patients. These treatable conditions are better targets for early detection than, say, all-cause mortality within the following year^28^, which has no clinical urgency, or ventricular fibrillation in the Coronary Care Unit, which has no prophylactic therapy.

We note, though, that not all clinical deteriorations are of this kind. Some acute illnesses in the hospital are genuinely of sudden onset—vascular catastrophes like acute myocardial infarction, cerebrovascular accident, pulmonary embolism, or arrhythmias such as ventricular fibrillation. For them, we expect no prodromes and no opportunities for forewarning. In fact, the absence of premonitory changes in the continuous cardiorespiratory monitoring delimits the differential diagnosis of sudden clinical deterioration.

#### Perspective

We expect subclinical prodromes for some subacute potentially catastrophic illnesses, so predictive analytics monitoring has face validity as a means for early detection.

### Signatures of illness

We built on the earlier observations of reduced heart rate variability in infants with respiratory distress^29–31^. In premature infants with late-onset sepsis at the University of Virginia, we saw something new. Transient decelerations, many of them too small to generate a bradycardia alarm, punctuated the otherwise unvarying heart rate record^7^. This is the same abnormality that distressed fetuses display^32,33^, and it is perhaps not, after all, surprising that premature infants might report illness in the same way. This signature of acute neonatal illness applies not just to sepsis but also to necrotizing enterocolitis^34^, respiratory distress, and bleeding^19^.

Note that this illness signature requires continuous cardiorespiratory monitoring to detect. It is not apparent by glancing at bedside monitors, nor is it captured in the EHR. Beaulieu-Jones and coworkers made the seminal observation that much of the predictive nature of the EHR lay in the orders placed by physicians on the day of admission^5^. They pointed out that such clinician-initiated actions reflected the thinking of the physicians rather than findings from the patients, and thus that EHR-only-based statistical models might be lagging indicators rather than leading ones. Delays in recording vital signs^35^ and in reporting lab results further increase the lag.

We endorse their view that telemetric real-time physiological monitoring is a source of non-clinician-initiated information that is more likely to reflect the patient’s status. The notion resonates clinically—why would we not use the patient’s physiologic data to make decisions about his or her physiologic status? We know well how the autonomic nervous system collects information from throughout the body and fine-tunes the heart and lungs in response^36^, and a new body of knowledge points to the sophistication of regulation of the sinus node and the heartbeat by intrinsic mechanisms^37^.

While sensible to consider in any clinical setting, continuous cardiorespiratory monitoring data are rarely used in illness scoring systems^38^ despite adding information by themselves^7,19,22,23,39–41^ or by adding to the EHR^20,24,42–44^. While we went on to find in the NICU that lab tests and clinical findings added independent information^42,43,45^, we stand by the practice of always using continuous cardiorespiratory monitoring data wherever we find it in the hospital.

#### Perspective

Signatures of illness are better detected when we record the right signals, those that tell us more about the patient than the clinician. For this task, models that use continuous cardiorespiratory monitoring will always be better than those that don’t.

### Sound mathematical time-series analysis and statistical modeling

The existing tools of heart rate variability analysis did not serve to detect records with abnormal heart rate characteristics because the decelerations inflate the standard deviation^7^. We have used time-domain^7,10,46–48^, frequency-^49,50^ and wavelet-domain^51,52^, phase-domain^53^, nonlinear dynamical-domain^8,9^, and other mathematical tools^47,48,54^ to characterize the dynamics of the heart and lungs from bedside continuous cardiorespiratory monitoring. Our final strategy^11^ comprised the standard deviation to detect long records with only reduced heart rate variability; sample asymmetry^10^, new measures of the decelerations and accelerations; and sample entropy^8^, which here serves to capture the phenotype of flat baselines with spikes^9^.

These approaches have irrefutable mathematical foundations not subject to changing points of view. We note the promise of a comprehensive strategy of Fulcher and coworkers that they named highly comparative time-series analysis^55,56^. Our recent work points to a reduced set of calculations on heart rate and oxygen saturation time series that captures many facets of cardiopulmonary physiology in premature infants^57^.

These kinds of quantitative methods can reliably and reproducibly lead to the optimal development of features that relate physiologic dynamics to outcomes. Such mathematical approaches differ from point scores using thresholds picked by experts, such as the Apgar score^58^, Score for Neonatal Acute Physiology (SNAP)^59^, or the Sequential Organ Failure Assessment (SOFA)^60^ and its neonatal version^61^, APACHE, and others, all made problematic by the need for thresholds and dichotomization^62^.

To optimize combinations of predictors, we have used mainly logistic regression in our work. We know of the proliferation of other machine learning and the newer recurrent neural network approaches of Deep Learning. (Indeed, we used a neural network in our first work on neonatal heart rate analysis in 1994)^46^. While the newer approaches have revolutionized radiology with image analysis, we find no clear and consistent superiority of one method over another in this field of classifying the risk of patients from clinical data^63^. We posit that newer machine learning and deep learning approaches^64–73^ should complement rather than replace traditional statistical pattern recognition methods.

#### Perspective

Once armed with the right signals, we should exercise the right analytic methods to quantify what they are telling us, ones that assay the physiological dynamics of the patient.

### Ground truth cases in the training sets

Chart review by clinicians is the gold standard for identifying cases on which to train statistical models. This observation stands to reason clinically, and multiple studies have quantified the shortcomings of automated detection strategies for infection^74^. There are two—failure to include cases in the training set, and dilution of the training set by non-cases. The impact depends on how the sensitivity and positive predictive accuracy compare to the incidence rate of the event. Say a good computer strategy for identifying events from the medical records has 70% sensitivity and 70% positive predictive accuracy^75^, but the event rate is only 1%. In that case, a study of 10,000 patients identifies 70 of the 100 events, reducing the richness of the training set, and includes 30 patients without the event, diluting the training set by nearly half with irrelevant cases. In addition to concerns about the robustness and precision of models trained on impure data sets, the new focus on explainability is endangered^70,76^. Confusion will follow when trying to understand the attributes of patients who did not have the targeted condition and failing to identify the attributes of those who did.

#### Perspective

Predictive models trained on all the actual cases and no others will always be better than those that aren’t.

### Dynamics of the model that match the course of the illness

While statistical testing of the performance of the heart rate characteristics index was essential^77^, there should be more to it than threshold-based evaluations like sensitivity and specificity or even areas under curves that evaluate multiple thresholds. (When a patient says s/he feels unwell, do you ask about their predictive performance?) We find that inspecting the time course of the model prediction as a function of the time until the event tells us much about what clinicians would see at the bedside. The phenotypes of the trajectories can say a great deal about the patient’s prognosis. For example, we identified trajectories of heart rate characteristics monitoring that differentiated septic patients into higher and lower risk categories^78^, a result presaged as long ago as 2003^12^. Indeed, it is often the trend over time more so than the magnitude of the risk that leads clinicians to act^79,80^.

While highly problematic statistically^62^, alerts based on threshold-crossings are not without value. The field of predictive analytics monitoring was recently advanced by Escobar and coworkers at Kaiser-Permanente who broadly adopted a very successful systems approach of alerts and informed intermediaries to reduce mortality at 19 hospitals^81^. But the problems of alert fatigue are well-known, and few risk estimates have true thresholds, where the risk steps up but is constant on either side of the breakpoint.

#### Perspective

Illnesses are dynamic, and the risk estimate should dynamically rise as the signature becomes more clear.

### A large randomized clinical trial

While RCTs have been criticized for expense, failure of scope, and limited applicability to clinical practice^82^, the design remains inarguably persuasive. While new designs are welcome^83^, the individually randomized trial remains a gold standard required to alter practice for many clinicians. The trial results overcome questions about metrics such as sensitivity and specificity and are antidotes to anecdotal reports.

For example, there were important reassurances in the heart rate characteristics trial about the possibility of increased sepsis work-ups. To be sure, since the event is rare, most positives are false^84^, and a review of a small subset of heart rate characteristics scores from one center had a negative conclusion^85^. We found, though, no significant increase in blood cultures or antibiotics^6^. We can surmise that low-risk scores must have averted about as many sepsis work-ups and rule-outs as high scores initiated, an opinion voiced by practitioners in the study^80^. This property of predictive analytics monitoring to reassure clinicians about the low-risk patients as well as to alert them to the high-risk ones is an additional utility not contemplated initially but emphatically present in the statistical analysis^77^.

#### Perspective

Randomized clinical trials of predictive analytics monitoring in the real world remain of premium value. Unless repeated, there can be no gainsaying the result.

## Current and future directions

A new area of work is implementing and integrating predictive analytics monitoring into the complex arena of clinical care. We note a bare-bones education in the neonatal ICU and an organic spread of its use mainly driven by word of mouth^80^. Our current implementation efforts in adult ICUs and wards of two hospitals and an eICU employ a systematic and principled approach^86^, and we note the applicability of the monitoring to a learning health systems approach^79^. Another new area is algorithmic equity. We propose that continuous cardiorespiratory monitoring may be less biased and less vulnerable to data shifts than the EHR as a data source, though work remains to test the ideas. The interpretability of models is another desirable feature^70^. We found that physicians and other clinicians wish to know the origins of rising risk as estimated by computers^79,86^. Finally, we anticipate studies of the utility of Deep Learning on the continuous cardiorespiratory monitoring time-series data, where new patterns undetected by domain experts might yet be found.

## Limitations of predictive analytics monitoring

Statistical models do not make diagnoses or tell us what to do next—all they can do is relate data to probability. It stands to reason that more data in more dimensions will improve the risk estimate, especially if the sampling is continuous, like bedside cardiorespiratory monitoring. Barriers to universal monitoring of hospital patients include the cost and the cumbersome nature of the devices. Several trends may change this picture. The pandemic has threatened the number of bedside clinicians who now serve to monitor patients closely, and technological advances have resulted in remarkably capable wearable devices that serve as cardiorespiratory monitors. Some day, perhaps one may need only to put an app on a watch to benefit from predictive analytics and other forms of continuous monitoring in the hospital.

Here is a more critical limitation: the data collected may not accurately paint the clinical picture of the patient. Like pointillism, a larger number of data points, and more strategically placed ones, better capture the identity of the illness. For a given patient, different clinicians might order different tests if their differential diagnoses differ. Each of the resulting data sets partially captures a competing view of the patient, further complicating the problem of making a statistical model for the classification of future patients. In the worst-case scenario, if a patient has sepsis but the chart has no recorded vital signs, labs, or other relevant data, then no scoring system can make an assessment. Beam and coworkers recently addressed the scenario when the predictive model has nothing to say on the matter^87^. A potential limitation of predictive analytics monitoring is that an irrelevant EHR record cannot assess the patient in the present, let alone for the future.

## Conclusion

We began our predictive analytics monitoring work more than 20 years ago by focusing on neonatal sepsis, a common and deadly illness with a subclinical prodrome and a signature of illness in continuous cardiorespiratory monitoring. We used mathematics to analyze non-clinician-initiated data in ground truth cases. The population- and illness-specific predictor changed dynamically with the risk of imminent illness, and its use improved outcomes in a large randomized trial. We believe that heart rate characteristics monitoring for neonatal sepsis is the earliest success of predictive analytics monitoring for subacute potentially catastrophic illness.

We offer this perspective as the template for our ongoing predictive analytics monitoring research, development, and implementation throughout the hospital. The guiding principles call for continuous cardiorespiratory monitoring, predictive models tailored for conditions and populations rather than just one model for the whole hospital, models trained on clinician-identified cases, sound mathematical foundations, display of changing risks rather than sounding alarms and alerts, and detailed schemes for implementation and integration that meld the predictive monitoring into the complex world of the hospital bedside.

### Reporting Summary

Further information on research design is available in the Nature Research Reporting Summary linked to this article.

## Supplementary information

Reporting summary checklist
